# Peroxisome proliferator-activated receptors, farnesoid X receptor, and dual modulating drugs in hypertension

**DOI:** 10.3389/fphys.2023.1186477

**Published:** 2023-06-23

**Authors:** John D. Imig

**Affiliations:** Department of Pharmaceutical Sciences, University of Arkansas for Medical Sciences, Little Rock, AR, United States

**Keywords:** blood pressure, proliferator-activated receptors, farnesoid X receptor, soluble epoxide hydrolase, diabetes

## Abstract

Hypertension characterized by an elevated blood pressure is a cardiovascular disease that afflicts greater than one in every three adults worldwide. Nuclear receptors are large superfamily of DNA-binding transcription factors that target genes to regulate metabolic and cardiovascular function. Drugs have been developed for nuclear receptors such as peroxisome proliferator-activated receptors (PPARα and PPARγ) and farnesoid X receptor (FXR). PPARα, PPARγ, and FXR agonists are used clinically to treat lipid disorders and metabolic diseases. Evidence from clinical studies and animal hypertension models have demonstrated that PPARα, PPARγ, and FXR agonism can lower blood pressure and decrease end organ damage which could be useful for the treatment of hypertension in patients with metabolic diseases. Unfortunately, PPAR and FXR agonists have unwanted clinical side effects. There have been recent developments to limit side effects for PPAR and FXR agonists. Combining PPAR and FXR agonism with soluble epoxide hydrolase (sEH) inhibition or Takeda G protein receptor 5 (TGR5) agonism has been demonstrated in preclinical studies to have actions that would decrease clinical side effects. In addition, these dual modulating drugs have been demonstrated in preclinical studies to have blood pressure lowering, anti-fibrotic, and anti-inflammatory actions. There is now an opportunity to thoroughly test these novel dual modulators in animal models of hypertension associated with metabolic diseases. In particular, these newly developed dual modulating PPAR and FXR drugs could be beneficial for the treatment of metabolic diseases, organ fibrosis, and hypertension.

## Introduction

Nuclear receptors are abundantly expressed in tissues that can influence blood pressure including the brain, vasculature, liver, and kidney (Oyekan, 2011; Usuda and Kanda, 2014). They represent a large superfamily of DNA-binding transcription factors that target genes critical for regulating biological processes (Usuda and Kanda, 2014). This review will focus on peroxisome proliferator-activated receptors (PPARs) and farnesoid X receptor (FXR) which have been implicated in hypertension, metabolic diseases, and end organ damage (Jones et al., 2021; Mori, et al., 2022; Rausch, et al., 2022; Hernández-Valdez et al., 2023). PPARs play an essential role in the regulation of diseases including dyslipidemia, obesity, diabetes, and hypertension (Jones et al., 2021; Hernández-Valdez, et al., 2023). There are three PPAR subtypes: PPARα, PPARγ, and PPARβ/δ, that have different organ expression patterns and distinct functions (Bookout, et al., 2006; Usuda and Kanda, 2014). The localization of PPARs to the vasculature, kidney, and brain have been linked to regulation of blood pressure and hypertension (Pavlov, et al., 2010; Fang, et al., 2021; Wang, et al., 2023). Another nuclear receptor that influences endothelial, kidney, heart, and metabolic function which could impact blood pressure regulation is the farnesoid X receptor (FXR) (Ishimwe, et al., 2022). FXR is a nuclear receptor, that is, activated by bile acids (Lin, et al., 2023). The main function for FXR is regulating bile acid synthesis, conjugation, and transport, as well as lipid and glucose metabolism (Mori, et al., 2022; Rausch, et al., 2022). Emerging evidence demonstrates that FXR agonism can have positive actions on metabolic diseases and chronic kidney diseases (Jiao, et al., 2022; Kim, et al., 2023). The fact that metabolic and kidney diseases impact blood pressure regulation provide impetus for exploring PPAR and FXR manipulation to combat hypertension associated with metabolic and kidney diseases.

Hypertension is the most prevalent cardiovascular disease that afflicts greater than thirty percent of the adult population (Oparil, et al., 2018; Mills et al., 2020). Although there are several drugs that treat hypertension, a large majority of adults with hypertension require more than one anti-hypertensive drug to control blood pressure (Oparil, et al., 2018). In addition, there is a large patient population that have an elevated blood pressure despite taking three and four anti-hypertensive drugs (Shalaeva and Messerli, 2023). Further complicating hypertension treatment is the significant portion of the population, that is, obese with metabolic diseases (Usui, 2023). The rise in obesity and type 2 diabetes along with the subsequent kidney damage contribute to the increasing incidence of hypertension (Usui, 2023). For this reason, dual modulating drugs that combat kidney and metabolic diseases are being explored for blood pressure lowering activity.

**FIGURE 1:**
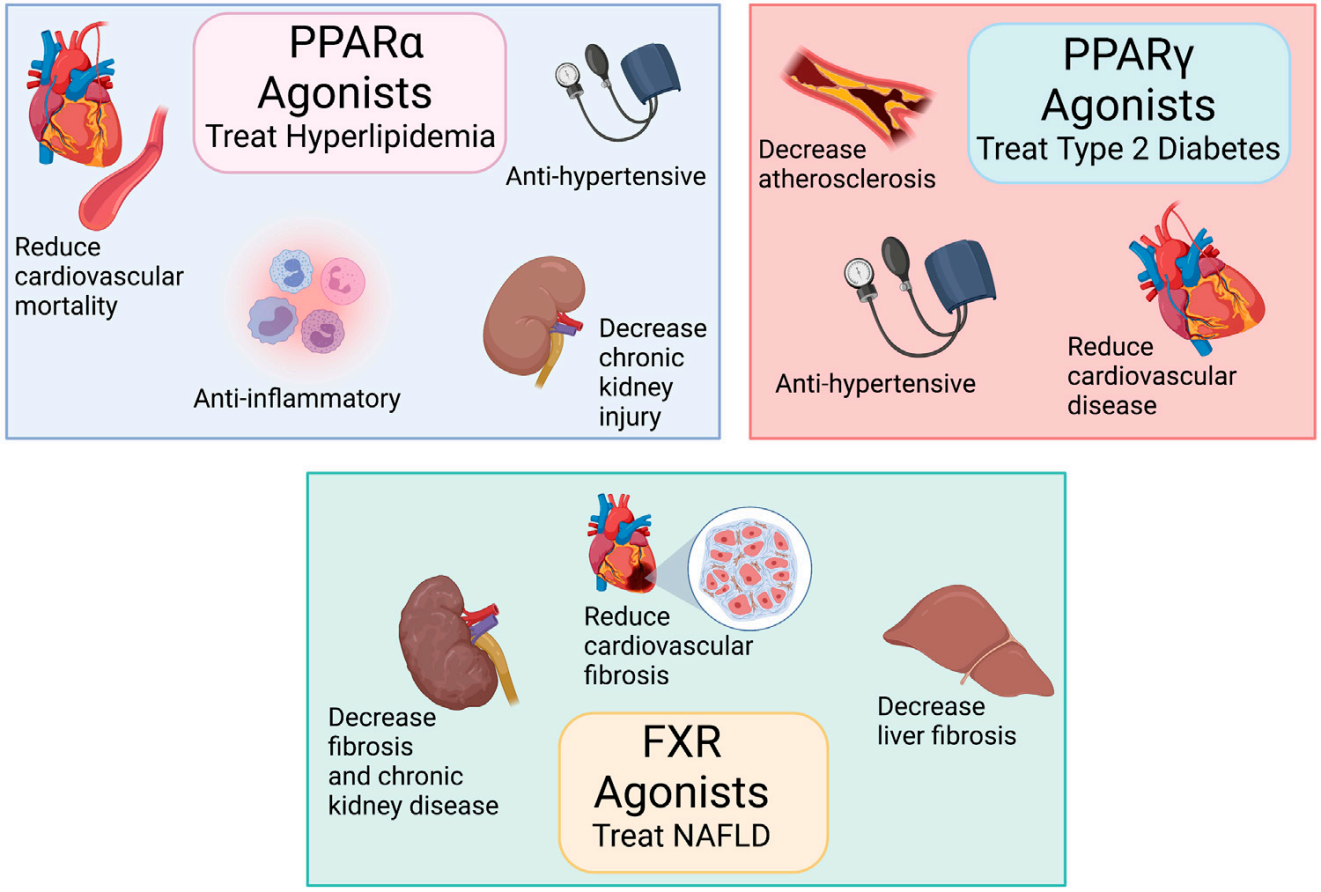
Peroxisome proliferator-activated receptors (PPARα and PPARγ) and farnesoid X receptor (FXR) agonists treat metabolic diseases: Top left panel: PPARα agonists treat hyperlipidemia, have anti-inflammatory actions, decrease cardiovascular diseases, and reduce chronic kidney disease. Top right panel: PPARγ agonists treat type 2 diabetes and reduce cardiovascular diseases. Bottom middle panel: FXR agonists treat non-alcoholic fatty liver disease (NAFLD) and reduces organ fibrosis. Created with BioRender.com.

PPAR and FXR agonism have been used to treat metabolic diseases; however, their use has been limited by clinical side effects (Lalloyer and Staels, 2010). PPARα agonists, fibrates, were approved for human use several decades ago for the treatment of dyslipidemia. Fibrates are still widely prescribed and exhibit additional pleiotropic to improve endothelial function, decrease myocardial ischemic, and combat immune-inflammatory responses (Lalloyer and Staels, 2010; Jao, et al., 2019) (Figure 1). Although clinical use of fibrates is safe, the most common adverse effects of this drug class is worsening liver function (Lalloyer and Staels, 2010; Usuda and Kanda, 2014). Intriguingly, despite these reports of blood pressure lowering actions for fibrates their use in hypertension is limited (Lalloyer and Staels, 2010; Gilbert, et al., 2013).

PPARγ activation by thiazolidinediones (TZDs) such as rosiglitazone and pioglitazone induce beneficial effects on insulin action and blood-glucose levels (Lalloyer and Staels, 2010; Usuda and Kanda, 2014). PPARγ is implicated both in several vascular conditions such as atherosclerosis and hypertension and TZDs lower blood pressure in humans and exert protective vascular effects through largely unknown mechanisms (Lalloyer and Staels, 2010; Usuda and Kanda, 2014; Fang, et al., 2021) (Figure 1). Unfortunately, the clinical use of TZDs is limited because of excessive weight gain, fluid retention, and increased risk of osteoporosis in treated patients (Lincoff et al., 2007; Lalloyer and Staels, 2010). There is a black box warning for TZD use in heart failure because treatment with rosiglitazone led to an increase in cardiovascular events (Yen, et al., 2021).

FXR agonists have been shown to be effective anti-fibrotic agents in liver and kidney disease animal models (Wang, et al., 2009; Zhang, et al., 2009). Moreover, FXR agonists have complex effects on glucose and lipid metabolism as well as on inflammation (Rausch, et al., 2022). (Figure 1) FXR has been examined as a target for non-alcoholic fatty liver disease (NAFLD) by the efficacy of obeticholic acid (OCA) in clinical trials (Shen, et al., 2021). The drug has revealed clinical side-effects specific to FXR signaling. OCA caused disturbances in cholesterol homeostasis with increased total cholesterol levels and a decrease in the HDL-c/non-HDL-c ratio in clinical trials (Mudaliar, et al., 2013; Neuschwander-Tetri, et al., 2015). This could be resolved by partial FXR activation, which appears to be a suitable approach to treat diseases such as hypertension associated with metabolic diseases while avoiding this putative FXR mechanism-based side-effect.

In this mini review we will detail the contributions of PPAR and FXR nuclear receptors to blood pressure regulation and hypertension. A second focus of the review will be on the emergence of dual modulating PPAR and FXR drugs for the treatment of hypertension associated with kidney and metabolic diseases.

## PPARs and FXR in hypertension

Nuclear receptors are targeted for metabolic diseases because of their importance in controlling energy homeostasis and inflammation. PPARα, PPARγ, and FXR agonists are used clinically to treat dyslipidemia, liver disease, and diabetes (Lalloyer and Staels, 2010; Shen, et al., 2021). The fact that hypertension exist in combination with these metabolic diseases has led to the findings that PPAR and FXR agonists can improve vascular function and lower blood pressure (Lalloyer and Staels, 2010; Fang, et al., 2021). These findings support the postulate that nuclear receptor PPARα, PPARγ, and FXR agonists have anti-hypertensive actions.

PPARα agonists, such as fibrates, are hypolipidemic drugs with anti-inflammatory actions that decrease kidney and cardiovascular complications in type 2 diabetes (Lalloyer and Staels, 2010). Fibrates have been demonstrated in animal studies to mediate their responses through activation of the PPARα receptor (NR1C1) which is highly expressed in the liver, kidney, and heart (Pawlak, et al., 2015). Clinical studies have determined that chronic treatment with fenofibrate decreases interleukin-6 (IL-6) to decrease atherosclerosis (Okopień, et al., 2005). In addition, fenofibrate treatment can lower intercellular adhesion molecule-1 (ICAM-1) and vascular cell adhesion molecule-1 (VCAM-1) in endothelial cells (Ryan, et al., 2007). Human outcome trials have demonstrated that PPARα agonists reduce cardiovascular morbidity in diabetes and metabolic syndrome (Staels, et al., 2008). PPARα activation decreases renal lipotoxicity, fibrosis, and inflammation in animal models of chronic kidney disease (Tanaka, et al., 2011; Jao, et al., 2019). Likewise, PPARα agonists lower blood pressure in animal models of hypertension (Roman, et al., 1993; Wilson, et al., 1998). Clinical studies data also demonstrate that fibrates reduce blood pressure in salt-sensitive hypertension (Gilbert, et al., 2013). The anti-hypertensive actions for PPARα agonists in hypertensive rats have been attributed to reduced Na^+^-K^+^ ATPase activity in the proximal tubules resulting in increased Na^+^ excretion (Wilson, et al., 1998). Proximal tubule PPARα attenuates renal fibrosis and inflammation caused by unilateral ureteral obstruction (Li et al., 2013). Animal studies have also demonstrated that PPARα activation can induce endothelial nitric oxide synthase (eNOS) and oppose endothelin vasoconstriction (Goya, et al., 2004). The PPARα activator fenofibrate increases renal endothelial hyperpolarizing factor and improves endothelial dilator function in obese Zucker rats (Zhao, et al., 2006). In addition, PPARα activation decreases IL-6 to reduce blood pressure in an animal model of angiotensin hypertension (Wilson, et al., 2012). Overall, PPARα agonists have hypolipidemic, vascular, and anti-inflammatory actions that could contribute to blood pressure lowering in hypertension.

PPARγ agonists, TZDs, are sensitize cells to insulin and improve insulin sensitivity to treat type 2 diabetes (Lalloyer and Staels, 2010). Pioglitazone and rosiglitazone are two commonly prescribed TZDs used in type 2 diabetes treatment. PPARγ has also been implicated in the maintenance of vascular homeostasis and cardiovascular diseases like atherosclerosis, hypertension, and restenosis (Lalloyer and Staels, 2010; Fang, et al., 2021). TZDs improve endothelial function and lower blood pressure in animal models of hypertension (Kvandová et al., 2016; Fang et al., 2021). Preclinical studies have demonstrated that activation of PPARγ improves endothelial function through decreased oxidative stress and increased nitric oxide availability (Majithiya et al., 2005; Kvandová et al., 2016; Fang et al., 2021). Likewise, PPARγ activation inhibits adhesion molecules to prevent vascular inflammatory damage (Straus and Glass, 2007; Fan et al., 2008). Liver injury associated with renal ischemia reperfusion injury in rodents is rescued by the PPARγ agonist, pioglitazone (Elshazly and Soliman, 2019). Preclinical studies have demonstrated that PPARγ agonism prevents TGF-β induced renal fibrosis by repressing EGR-1 and STAT3 (Németh et al., 2019). These vascular anti-inflammatory and organ protective actions of PPARγ improve vascular function in patients with atherosclerosis and hypertension with or without diabetes (Patel et al., 2006; Lalloyer and Staels, 2010). The ability for TZDs to lower blood pressure in humans could include suppression of the renin-angiotensin system and inhibition of the angiotensin type 1 (AT1) receptor (Lalloyer and Staels, 2010; Fang et al., 2021). These findings support the notion that PPARγ agonists can potentially treat hypertension independent of their anti-diabetic actions.

Although FXR regulates bile acids and lipid and glucose metabolism, FXR is expressed in the kidney and FXR agonism has beneficial effects to prevent kidney inflammation and fibrosis in disease animal models (Jiao et al., 2022; Kim et al., 2023). In addition, in the unilateral ureter obstruction (UUO)-induced kidney fibrosis mouse model, FXR agonism reduces tubulointerstitial fibrosis by decreasing TGFβ-Smad3 signaling (Li et al., 2019). Kidney injury in uninephrectomized obese mice is also reduced by FXR agonism (Gai et al., 2016). FXR agonism modulates renal lipid metabolism, fibrosis and decreases diabetic nephropathy in rodents (Jiang et al., 2007; Marquardt et al., 2017). The mechanism for decreasing renal fibrosis appears to be dut to Src-mediated crosstalk between FXR and YAP (Kim et al., 2019). FXR agonists have also been demonstrated to lower blood pressure in spontaneously hypertensive rats (SHR) and mice with salt-sensitive hypertension (Li et al., 2015; Zhu et al., 2022). The promise of FXR activation to treat cardiovascular and kidney fibrosis is dampened by unwanted increases in cholesterol levels in humans (Mudaliar et al., 2013; Neuschwander-Tetri et al., 2015). Dual modulating drugs with FXR agonism have avoided this problem and are demonstrating promising actions to combat kidney and cardiovascular diseases (Miyazaki-Anzai et al., 2010; Stavniichuk et al., 2020).

## Development of dual modulating drugs with PPAR and FXR agonism

Interest in dual modulating drugs has intensified over the past decade and gained traction for the treatment of complex diseases (Jhund and McMurray, 2016; Imig et al., 2021; Lillich et al., 2021). Advantages of dual modulating drugs include complex disease modifying actions, synergistic therapeutic properties, predictable pharmacokinetics, and decreased drug interactions (Imig et al., 2021). Preclinical studies have demonstrated that the dual PPARα/PPARγ agonist, tesaglitazar, improves metabolic abnormalities and reduces renal injury in obese Zucker rats (Liao et al., 2010). Intriguingly, several AT1 receptor blockers act as dual modulating drugs by possessing PPARγ affinity (Taguchi et al., 2011). PPARγ and klotho mediate the renal protective effect of Losartan in 5/6 nephrectomy model (Maquigussa et al., 2018). The AT1 receptor blocker, telmisartan, has partial PPARγ modulatory activity; however, clinical trials in humans were inconsistent with regards to beneficial glucose and lipid metabolism actions (Fogari et al., 2009; Wago et al., 2010; Hamada et al., 2014; Naruse et al., 2019). Telimisartan has relatively weak PPARγ potency; however, this has been used as the starting point for developing sartans with greater PPARγ modulatory activity (Choung et al., 2018; Lillich et al., 2021). These findings of beneficial metabolic effects with telmisartan supported the notion that combining an anti-hypertensive action and nuclear receptor activation could be effective in the treatment of cardiovascular diseases associated with metabolic disorders.

**FIGURE 2:**
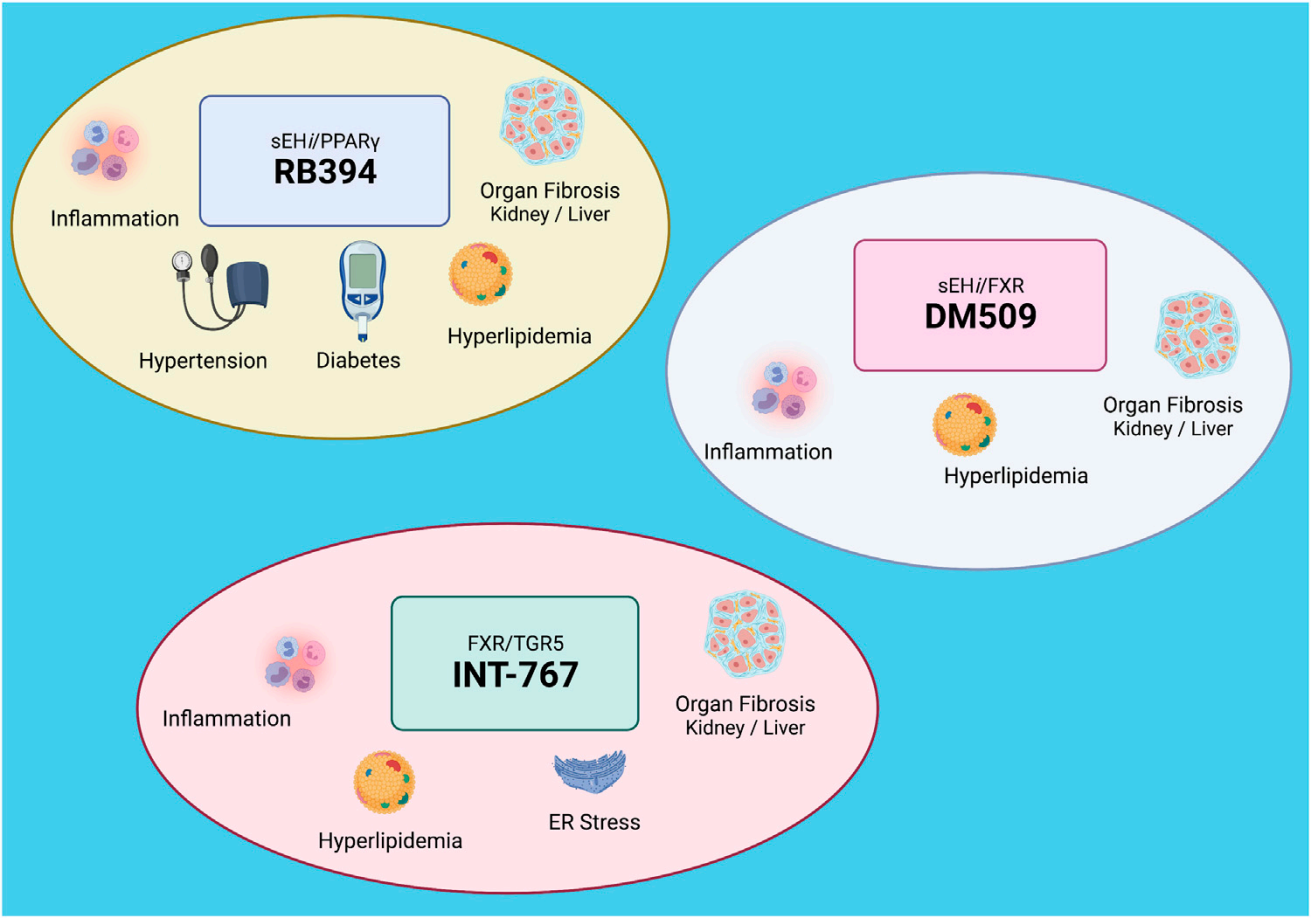
Dual modulating drugs combat metabolic diseases: Top left: RB394 is a dual modulating soluble epoxide hydrolase inhibitor (sEH*i*) and peroxisome proliferator-activated receptor gamma (PPARγ) agonist that combats hypertension and diabetes and decreases inflammation, hyperlipidemia, and organ fibrosis. Bottom left: INT-767 is a dual modulating farnesoid X receptor (FXR) and Takeda G protein receptor 5 (TGR5) agonist that decreases inflammation, hyperlipidemia, endoplasmic reticulum (ER) stress, and organ fibrosis. Top right: DM509 is a dual modulating sEHi and FXR agonist. DM509 decreases inflammation, hyperlipidemia, and organ fibrosis. Created with BioRender.com.

The anti-inflammatory, anti-fibrotic, anti-hypertensive, and anti-diabetic actions of drugs that act on arachidonic metabolism have been combined with nuclear receptor agonists (Lillich et al., 2021). Drugs that act on the arachidonic acid soluble epoxide hydrolase (sEH) enzyme combined with PPARγ and FXR agonism have been developed to treat cardiovascular diseases, diabetes, and kidney diseases (Imig et al., 2021; Lillich et al., 2021). Inhibition of sEH prevents the hydrolysis of arachidonic acid metabolites, epoxyeicosatrienoic acids (EETs), to their corresponding, less bioactive diols (Imig, 2012). EETs increase sodium excretion, dilate blood vessels, and oppose inflammation to lower blood pressure in animal disease models (Imig, 2012). Through sEH inhibition, EET levels are increased in animals and humans (Imig, 2012). A dual acting PPARγ agonist and sEH inhibitor (RB394) has demonstrated in preclinical studies to lower blood pressure, reduce kidney injury, and treat diabetic complications (Hye Khan et al., 2018). (Figure 2) RB394 is a merged pharmacophore multi-target drug that engages both biologic targets of interests at submicromolar potency (Imig et al., 2021). RB394 has been evaluated in the spontaneously hypertensive obese rat and the obese diabetic ZSF1 rat models (Hye Khan et al., 2018). Spontaneously hypertensive obese rats treated with RB394 had reduced blood pressure, improved insulin sensitivity, lower plasma lipids, and decreased kidney injury (Hye Khan et al., 2018). RB394 treatment to obese diabetic ZSF1 rats for 2 months reduced blood glucose levels, improved glucose tolerance, reduced blood pressure, and improved lipid profiles (Hye Khan et al., 2018). Interestingly, RB394 ameliorated liver fibrosis, hepatosteatosis, and diabetic nephropathy in obese diabetic ZSF1 rats (Hye Khan et al., 2018). Importantly, RB394 does not lead to excessive weight gain or fluid retention associated with PPARγ agonist TZDs (Hye Khan et al., 2018). Likewise, the eutomer of RB394 promotes adipocyte browning in cell culture and exhibits cardioprotective activity in isolated perfused mice hearts following ischemia (Hartmann et al., 2021). Thus, the combination of sEH inhibition and PPARγ agonism reduces side effects while increasing efficacy for hypertension and diabetes.

The combination of sEH inhibition and FXR agonism have been developed and tested for beneficial actions for nonalcoholic steatohepatitis (NASH) and kidney fibrosis (Hye Khan et al., 2019; Stavniichuk et al., 2020). The dual sEH inhibitor and FXR agonist, DM509 potently inhibits sEH while partially activating FXR (Schmidt et al., 2017). Partial FXR activation in DM509 allows for the exploitation of the beneficial effects of FXR activation while avoiding marked effects on cholesterol metabolism (Schmidt et al., 2017; Hye Khan et al., 2019). (Figure 2) Evaluation of DM509 in two liver fibrosis models demonstrated anti-fibrotic activity that was greater than the FXR agonist OCA (Schmidt et al., 2017; Hye Khan et al., 2019). Importantly, DM509 administered to NASH mice lowered triglyceride levels and increased the HDL-cholesterol/non-HDL-cholesterol ratio (Schmidt et al., 2017). Likewise, experimental studies in UUO mice determined that DM509 decreased renal fibrosis through anti-inflammatory actions (Stavniichuk et al., 2020). The ability for DM509 to have blood pressure lowering actions and decrease cardiovascular injury has yet to be determined.

Another dual modulating FXR agonist is INT-767 where FXR agonism is combined with Takeda G protein receptor 5 (TGR5) agonism (Rizzo et al., 2010). TGR5 agonism leads to dependent glucagon-like peptide-1 (GLP-1) secretion by enteroendocrine cells which can combat metabolic diseases (Rizzo et al., 2010; Jadhav et al., 2018). INT-767 is a dual FXR and TGR5 agonists that selectively activates both bile acid receptors and fails to activate any other nuclear receptor or G protein couple receptor tested (Rizzo et al., 2010). Treatment with INT767 to metabolic disease animal models resulted in beneficial metabolic and liver effects (Comeglio et al., 2018; Wang et al., 2018). (Figure 2) These positive metabolic actions for INT-767 impact brown adipogenesis and mitochondrial function (Comeglio et al., 2018). INT-767 also improves plasma cholesterol and triglyceride levels and reduces kidney injury in obese and diabetic mice (Wang et al., 2018). Cell signaling pathways impacted by INT-767 include AMP-activated protein kinase, sirtuins, PGC-1α, and nuclear respiratory factor 1 which decrease endoplasmic reticulum stress (Wang et al., 2018). INT-767 also reduces inflammation and mitochondrial function to decrease glomerular injury in mice (Wang et al., 2017). There is a high therapeutic potential for INT-767 to treat metabolic and kidney diseases; however, it remains to be determined if dual FXR and TGR5 agonism have beneficial actions in hypertension.

## Discussion

PPAR and FXR agonism has been used to treat lipid disorders and metabolic disease (Lalloyer and Staels, 2010; Rausch et al., 2022). Clinical and experimental data have revealed that FXR and PPAR agonism could also lower blood pressure in hypertension (Lalloyer and Staels, 2010; Li et al., 2015; Zhu et al., 2022). There has been a recent focus on combining PPAR and FXR agonism with sEH inhibition or TGR5 agonism to combat metabolic diseases and organ fibrosis (Rizzo et al., 2010; Lillich et al., 2021). Initial preclinical studies with the dual acting PPARγ agonist and sEH inhibitor, RB394, has been demonstrated to lower blood pressure and improve cardiovascular function (Hye Khan et al., 2018). There is still a need to develop and evaluate additional dual modulating drugs that include TGR5, PPARα, PPARγ, FXR agonism, sEH inhibition, and other activities. Although dual modulating drugs are designed to treat complex diseases while minimizing side effects, these drugs will still need to be assessed for adverse effects. These novel dual modulators would need to be tested in animal models of metabolic disease and hypertension. Ultimately, there is great promise for dual modulating PPAR and FXR drugs for the treatment of metabolic diseases, organ injury, and hypertension.
